# Comparing individual-, family-, and community-level effects on the oral health of preschool children: a multilevel analysis of national survey data

**DOI:** 10.1186/s12903-023-03077-w

**Published:** 2023-06-02

**Authors:** Ying-Chun Lin, Shun-Te Huang, Cheng-Wei Yen, Yung-Kai Huang, Tzong-Ming Shieh, Wei-Hsueh Chi, Wu-Lin Yao, Pei-Shan Ho

**Affiliations:** 1 Department of Oral Hygiene, Shu-Zen College of Medical and Management, Kaohsiung, Taiwan; 2 Division of Pediatric and Special Needs Dentistry, Department of Dentistry, Kaohsiung Medical University Hospital, Kaohsiung, Taiwan; 3 Department of Oral Hygiene, Kaohsiung Medical University, Kaohsiung, Taiwan; 4 School of Dentistry, China Medical University, Taichung, Taiwan; 5 School of Dentistry, College of Dental Medicine, Kaohsiung Medical University, Kaohsiung, Taiwan; 6 Division of Medical Statistics and Bioinformatics, Department of Medical Research, Kaohsiung Medical University Hospital, Kaohsiung Medical University, Kaohsiung, Taiwan

**Keywords:** Caries, Deciduous teeth, Contextual effect, Individual-level, Family-level, Community-level

## Abstract

**Background:**

Early childhood is a critical stage for the prevention of dental caries. The prevalence of caries in preschool children is still high in Taiwan, where National Health Insurance covers 99% of the population. The effort to improve the oral health of preschool children should be based on conceptual model that encompasses more than individual-level factors. This study input nationwide survey data in a conceptual model to evaluate the effects of comprehensive factors related to the high prevalence of caries in preschool children.

**Methods:**

This observation study examined factors related to the oral health of preschool children by employing a comprehensive multilevel model to analyse nationally representative data from the Taiwan Oral Health Survey of Preschool Children (TOHPC) 2017–2018. Individual-level, family-level and community-level contextual effects were evaluated through multilevel analysis in this study. The proportional change in variance (PCV) was used to compare the multilevel model with the null model and individual-level, family-level, and community-level context effects.

**Results:**

The estimated deft index for preschool children was 1.34 (1.22–1.47) at age 3, 2.20 (2.08–2.32) at age 4, and 3.05 (2.93–3.18) at age 5. The overall prevalence of caries in preschool children in Taiwan was 34.27% (30.76%, 37.78%) at age 3, 51.67% (48.99%, 54.35%) at age 4, and 62.05% (59.66%, 64.44%) at age 5. The model that included the individual-, family-, and community-context levels exhibited the highest reduction of variance (PCV = 53.98%). The PCV was further reduced to 35.61% when only the level of accessibility to dental services for individuals, families, and the community was considered. For the model in which no community-context cofactors were considered and the model considering only the individual level, the PCVs were 20.37% and 5.52%, respectively.

**Conclusions:**

Our findings indicate the key components that affect oral health in preschool children and can serve as a reference for policy makers. The most notable finding of this study is that to improve the oral health of preschool children, community-level factors should be targeted. To rely solely on dentists for leading oral health education programs for children is impractical and inefficient. Training more professional oral health educators to provide additional community-based oral health promotion campaigns is critical. We suggest training more professional oral health educators to provide more community-based oral health promotion campaigns.

## Introduction

Dental caries are one of the most prevalent chronic diseases worldwide and a highly prevalent health problem in children. Despite the declining prevalence of caries reported by many countries, the problem persists in Taiwan. In a national survey of Taiwan in 2011, the prevalence of caries in 5-year-old children was more than 79.3%, which is higher than that of many other countries [1]. Early childhood is a critical stage for the prevention of caries [2]. The most valid predictive factor for experiencing caries in the permanent dentition is experiencing caries in the primary dentition [3].

Factors related to the presence of caries in preschool children include personal demographic characteristics, dietary and oral health habits, family socioeconomic status, parent and caregiver oral health knowledge, and oral healthcare resource accessibility [4–6]. Studies have indicated that a lack of dental services is a critical risk factor that negatively affects the oral health status of preschool children [7, 8]. However, the effect of the family on these children’s oral health is more pronounced than that of clinical services [7]. Because individual children live in families, and those families are embedded in communities, the community can shape parents’ knowledge, which enables them to impart beneficial oral hygiene habits to their children [2]. Therefore, the individual, family, and community levels should all be considered when determining which factors influence preschool children’s oral health and related resource use. The theoretical concept of multiple levels of health determinants was developed by Fisher-Owens [7], which is also recommended by the World Health Organization (WHO) [1]. Taiwan does not fluoridate its water supply, which contributes to a high prevalence of dental caries in preschool children. However, public policy related to the oral health of preschool children has improved in Taiwan in recent years; since 2004, the government has provided fluoride varnish application (FVA) to all preschool children. All children younger than 5 years old are eligible for the biannual dentist-administered FVA treatment. In addition to FVA, the treatment also includes clinical examination and oral health education for the parents or caregivers of preschool children. However, the utilisation rate of this service is not high, and 54.2% of eligible children did not receive FVA during the period of 2004–2013 [9]. Starting in 2014, molar pit and fissure sealant was also applied during the treatment of 6-year-old children. The high prevalence of caries and low utilisation of preventive services underscore the urgency of finding an effective strategy for improving the oral health of preschool children.

Determinants of oral health disparities represent a complex mix of the individual, family and community factors. Without considering social-related factors, policies for the prevention of caries will be less effective. This study applied a multilevel conceptual model to analyse population-based national survey data to determine the factors that contribute to the high prevalence of caries among preschool children; the importance of various aspects of each level of the conceptual model was investigated. We contend that this study will provide guidance for the design of more effective public policies for improving the oral health of preschool children.

There were two objectives in this study. The first objective is to explore the caries status of different demographic factors, such as age, gender, urban–rural disparity. The second one is to find the risk factors related to the caries status of preschool child. Among public health workers focused on oral health, a greater emphasis should be placed on population-based cofactors, especially on the oral health status of preschool children [7]. The greatest strength of this conceptual model is that it incorporates comprehensive dimensions related to the prevalence and prevention of caries in preschool children; additionally, its results can guide researchers in finding suitable prevention strategies. In sum, this study input nationwide survey data in a conceptual model to evaluate the effects of comprehensive factors related to the high prevalence of caries in preschool children.

## Material and methods

### Study design and sample selection

The Taiwan Oral Health Survey of Preschool Children (TOHPC) of 2017–2018 was a cross-sectional survey of the population segment under 6 years old. The TOHPC was funded by the Ministry of Health and Welfare of Taiwan. To select participants for the survey, a multiple-stage sampling design was implemented. National Health Insurance (NHI) in Taiwan covers 99% of the population, and dental treatment is also covered by NHI. Taiwan is subdivided into six NHI areas. Two counties (cities) were selected from each NHI area by probability proportional to size sampling (PPS), yielding a total of 12 counties (cities). For each selected county, we classified all districts (villages) into two levels, according to the socioeconomic status of their residents [10]. PPS was also utilised to select one representative district (village) for each of the two levels of socioeconomic status for each county. For every selected district (village), participants who were 3 to 5 years old were randomly selected from local kindergartens, childcare centres (public or private), or health clinics. Hence the participants are preschool children, the informed consent have been obtained from a parent and/or legal guardian of participants**.** In this study, an informed consent form was sent to the parents and/or legal guardian of the selected children, and those who returned the signed informed consent forms were invited to participate in the study. This project was reviewed and approved by the Institutional Review Board of Kaohsiung Medical University Hospital (KMUHIRB-SV(I)-20,170,017).

### Data collection and analysis methods

This study outcome was defined as the experience of dental caries, which was measured as the experience of dental caries on primary teeth (the deft index). Oral examinations were conducted according to the standards of the WHO Oral Health Surveys, 5^th^ edition, and performed by dentists who underwent standardization training for the examination protocol. The questionnaire for parents and caregivers was used to collect information regarding socio-demographic data, oral health behaviour, oral health knowledge, preventive oral health knowledge and preventive policy knowledge, and use of dental services. Socio-demographic data were collected to obtain an individual profile of each child (child’s gender, parent’s/ caregiver’s education). Oral health behaviour of children was asked about tooth brushing frequency, brushing timing and fluoride tooth paste use. Oral health knowledge, preventive oral health knowledge and preventive policy knowledge were based on sum of 13 items, 9 items and 7 items questionnaire separately. And the score of oral health knowledge, preventive oral health knowledge and preventive policy knowledge were quartile as no, low, median and high. Community level factors include the density of population and dentist, the density of area and dental clinic, average score of oral health and preventive knowledge in the community.

Basic demographic information is shown by frequency distribution. Associations between demographic characteristics and dental caries were analysed by performing the chi-square test. The nationally representative deft index was estimated according to age and sex with a 95% confidence interval. Three-level models were fitted for the primary outcome variables in SAS GLIMMIX (SAS Institute, Inc., Cary, NC, USA, 2006), which does not provide likelihood values for likelihood ratio tests; the fit statistics produced in GLIMMIX are pseudolikelihoods that should not be compared across models to determine model fit. We defined the caries variables as dichotomous dependent variables according to oral exam outcomes. A deft index score after oral examination equal to zero denotes ‘no caries’, and a deft index score larger than zero denotes the presence of caries. Data were weighted to adjust for varying probabilities of selection.

Multilevel analysis was conducted for five models. First, a null model was used to estimate the variance by considering only random effects. Model 1 included individual-level covariables, and model 2 included both individual- and family-level covariables. The last two models also included contextual covariables, which were the accessibility of community dental services for model 3 and the oral health–related knowledge status of the community for model 4. We examined the magnitude of community-level context effects using intraclass correlation coefficients (ICC), median odds ratios (MORs) and PCV to compare the multilevel models with the null model for individual-level, family-level, and community-level context effects. The data and analysis reported for the cross-sectional study follow the Strengthening the Reporting of Observation studies in Epidemiology (STROBE) guideline.

## Results

A total of 7491 preschool children were included in this study. In Table 1, the number of male children (*n* = 3870, 51.63%) was slightly higher than the number of female children (*n* = 3626, 48.37%). When comparing demographic characteristics with caries status, we found statistically significant higher prevalence rates for caries in children from rural areas (60.91%), for caries in children of parents with lower levels of education (68.16%), for caries in children who did not brush their teeth twice per day (59.40%), and for caries in children who did not brush their teeth before going to bed (63.64%). Children who habitually ate snacks or drank sweetened beverages also had higher prevalence rates for caries, with 59.99% and 62.90%, respectively. A lower prevalence of caries was found in the children with previous FVA treatment and a history of regular dental visits. The weighted caries index and caries prevalence rates are shown in Table 2. The estimated deft index for preschool children was 1.34 (1.22–1.47) at age 3, 2.20 (2.08–2.32) at age 4, and 3.05 (2.93–3.18) at age 5. The overall prevalence of caries in preschool children in Taiwan was 34.27% (30.76%, 37.78%) at age 3, 51.67% (48.99%, 54.35%) at age 4, and 62.05% (59.66%, 64.44%) at age 5. No statistically significant difference was evident between sexes at the same age.

**Table 1:** The oral health and dental visit status by the basic characteristic of children

	Total		Caries			
			No		Yes	
	N	%	N	%	N	%
**Age group**						*P*<0.0001
3	1527	(19.85)	875	(57.30)	652	(42.70)
4	2651	(34.46)	1152	(43.46)	1499	(56.54)
5	3318	(43.12)	1150	(34.66)	2168	(65.34)
**Gender**						*P*=0.6331
Male	3870	(50.299)	1630	(42.12)	2240	(57.88)
Female	3626	(47.128)	1547	(42.66)	2079	(57.34)
**Area**						*P*<0.0001
Rural	3832	(49.805)	1498	(39.09)	2334	(60.91)
Urban	3664	(47.622)	1679	(45.82)	1985	(54.18)
**Education level of parents**						*P*<0.0001
Both below college	1517	(19.717)	483	(31.84)	1034	(68.16)
One of parents below college	1778	(23.109)	670	(37.68)	1108	(62.32)
Both above college	4201	(54.601)	2024	(48.18)	2177	(51.82)

**Table 2:** The status of caries and regular visit status by age group

Gender	Age group	dft		Caries prevalence	
		mean	95%CI	rate	95%CI
Male	3	1.91	(1.69, 2.12)	43.81%	(40.42%, 47. 20%)
	4	2.72	(2.52, 2.92)	55.99%	(53.31%, 58.67%)
	5	3.54	(3.35, 3.73)	66.03%	(63.80%, 68.26%)
Female	3	1.69	(1.48, 1.90)	41.39%	(37.75%, 45.04%)
	4	2.75	(2.56, 2.95)	57.09%	(54.43%, 59.75%)
	5	3.33	(3.14, 3.52)	64.59%	(62.24%, 66.94%)
Total	3	1.81	(1.66, 1.96)	42.70%	(40.22%, 45.18%)
	4	2.74	(2.60, 2.88)	56.54%	(54.66%, 58.43%)
	5	3.44	(3.31, 3.57)	65.34%	(63.72%, 66.96%)

We present the effects of factors affecting caries status at the individual, family, and community levels determined through hierarchical logistic regression in Table 3. Four models are shown in this table. The first only examined individual factors, and the resulting ORs for caries in children aged 4 and 5 years were 1.83 (1.28, 2.6) and 2.57 (2.05, 3.22), respectively, when compared with children age 3. Habitual sweetened beverage drinkers had a significantly higher risk of caries (OR 1.52 [1.27, 1.82]). However, eating snacks and personal oral health behaviours were not found to affect the caries status of preschool children. In the second model, the individual and family levels were considered. Individual-level factors were not significantly affected by family-level factors. For family-level factors, preschool children whose parents or caregivers helped them brush their teeth every day had significantly lower risk of caries (OR 0.81 [0.71, 0.92]). Having parents or caregivers who had high preventive dentistry knowledge and higher levels of education (both parents with an education above the college level) provided a significantly greater protective effect for the prevention of caries (ORs 0.81 [0.70, 0.95] and 0.64 [0.44, 0.92], respectively). Having parents or caregivers with lower levels of self-assessed oral health was a risk factor for caries status in preschool children. Compared with having parents or caregivers with a high level of self-assessed oral health, the OR for having parents or caregivers with median and low levels of self-assessed oral health was 1.49 (1.28, 1.73) and 1.69 (1.38, 2.06), respectively. Models 3 and 4 also considered community-level factors. In model 3, the accessibility of dental services was considered a community-level effect, and the community effect for oral health and prevention knowledge were also accounted for in model 4. When compared with children living in communities with lower than average preventive dentistry knowledge, children living in communities with higher than average preventive dentistry knowledge had significantly lower prevalence of caries (OR 0.67 [0.45, 0.98]).

**Table 3:** Hierarchical logistics regression of the three level factors related to caries status

	Model 1				Model 2			Model 3		Model 4	
	**OR**			**95%CI**	**OR**	**95%CI**		OR	95%CI	**OR**	**95%CI**
**children level**											
Age group (ref=3)											
4	1.73			(1.24, 2.43)	1.75	(1.24, 2.47)		1.75	(1.42, 2.17)	1.76	(1.43, 2.18)
5	2.28			(1.83, 2.84)	2.30	(1.82, 2.90)		2.30	(1.75, 3.02)	2.32	(1.77, 3.05)
Gender (ref= female)											
male	1.02			(0.85, 1.22)	1.02	(0.85, 1.22)		1.02	(0.88, 1.18)	1.02	(0.88, 1.18)
Height	1.01			(0.99, 1.03)	1.01	(0.99, 1.03)		1.01	(0.99, 1.03)	1.01	(0.99, 1.03)
Weight	1.03			(0.99, 1.06)	1.02	(0.99, 1.06)		1.02	(0.98, 1.06)	1.02	(0.98, 1.06)
Tooth brushing >= 2/day(ref = No)											
Yes	1.01			(0.80, 1.28)	1.02	(0.80, 1.29)		1.02	(0.86, 1.21)	1.02	(0.86, 1.21)
Tooth brushing before going to bed(ref=No)											
Yes	1.06			(0.79, 1.42)	1.03	(0.79, 1.33)		1.03	(0.81, 1.31)	1.03	(0.81, 1.32)
Helping brushing everday (ref=No)											
Yes	0.71			(0.62, 0.80)	0.77	(0.67, 0.87)		0.77	(0.64, 0.91)	0.77	(0.65, 0.91)
Toothpaste (ref=No)											
Yes	0.96			(0.78, 1.18)	0.97	(0.81, 1.16)		0.97	(0.81, 1.17)	0.96	(0.80, 1.16)
Fluoride toothpaste (ref=No)											
Yes	1.02			(0.86, 1.21)	1.07	(0.88, 1.30)		1.07	(0.91, 1.26)	1.07	(0.91, 1.26)
Eat snacks more than twice every week (ref=No)											
Yes	1.04			(0.84, 1.29)	0.98	(0.80, 1.21)		0.99	(0.84, 1.15)	0.99	(0.84, 1.15)
Sweet beverage drinking (ref=No)											
Yes	1.44			(1.17, 1.76)	1.39	(1.15, 1.69)		1.39	(1.19, 1.63)	1.39	(1.19, 1.63)
Flouoride vanish (ref=No)											
Yes	0.72			(0.64, 0.81)	0.76	(0.66, 0.87)		0.76	(0.62, 0.92)	0.76	(0.62, 0.93)
Regular dental visit (ref=No)											
Yes	1.03			(0.77, 1.37)	1.13	(0.86, 1.49)		1.13	(0.95, 1.35)	1.14	(0.96, 1.36)
**Family level**											
Dental preventive policy knowledge (ref=No)											
Low					0.83	(0.57, 1.20)		0.82	(0.661.03)	0.82	(0.661.03)
Median					0.97	(0.69, 1.35)		0.97	(0.76, 1.24)	0.97	(0.76, 1.24)
High					1.01	(0.62, 1.65)		1.01	(0.74, 1.37)	1.01	(0.74, 1.38)
Dental preventive knowledge (ref=No)											
Low					0.99	(0.76, 1.29)		0.99	(0.78, 1.25)	0.99	(0.79, 1.25)
Median					1.01	(0.81, 1.26)		1.01	(0.84, 1.21)	1.02	(0.85, 1.22)
High					0.82	(0.67, 1.01)		0.82	(0.66, 1.01)	0.82	(0.67, 1.01)
Oral health knowledge (ref=No)											
Low					1.09	(0.83, 1.44)		1.09	(0.88, 1.35)	1.09	(0.88, 1.35)
Median					0.95	(0.77, 1.19)		0.95	(0.78, 1.17)	0.95	(0.78, 1.17)
High					0.96	(0.66, 1.37)		0.95	(0.76, 1.20)	0.96	(0.76, 1.20)
Self-efficiency(ref=low)											
Median					1.02	(0.67, 1.55)		1.02	(0.66, 1.58)	1.02	(0.66, 1.57)
High					0.82	(0.48, 1.40)		0.83	(0.54, 1.27)	0.82	(0.54, 1.26)
Education level of caregiver (ref=below college)											
Above college					0.94	(0.73, 1.21)		0.94	(0.74, 1.21)	0.94	(0.74, 1.21)
Caregivers' self-assessment of oral health (ref=High)											
Median					1.58	(1.35, 1.83)		1.57	(1.34, 1.85)	1.57	(1.34, 1.85)
Low					1.79	(1.43, 2.23)		1.79	(1.42, 2.25)	1.79	(1.42, 2.25)
Education level of parents (ref=both below college)											
One of parents below college					0.85	(0.66, 1.10)		0.85	(0.63, 1.14)	0.85	(0.63, 1.14)
Both above college					0.67	(0.47, 0.98)		0.67	(0.49, 0.92)	0.67	(0.49, 0.93)
**Community level**											
Ratio of population and dentist								1.37	(0.98, 1.90)	1.31	(0.96, 1.79)
Interval Odds Ratios (IOR)									(0.94, 1.99)		(0.98, 1.76)
Ratio of area and dental clinic								0.96	(0.64, 1.42)	0.81	(0.53, 1.23)
Interval Odds Ratios (IOR)									(0.66, 1.39)		(0.60, 1.09)
Mean of oral health knowledge in the community										1.08	(0.75, 1.55)
Interval Odds Ratios (IOR)											(0.80, 1.45)
Mean of dental preventive knowledge in the community										0.66	(0.46, 0.95)
Interval Odds Ratios (IOR)											(0.49, 0.89)

The random-effects model was used to represent the magnitudes of individual-level, family-level, and community-level contextual effects (Table 4)3. In the hierarchical structure, we found that the ICC and MOR values slightly decreased for the individual-, family-, and community-level adding sequences. The ICC and MOR represent variation between random factors (i.e. rural or urban community). The decrease in ICC and MOR indicate that variations in community type are reduced by considering other level factors. The MOR in each model was larger than 1, indicating that unexplained cluster heterogeneity (comparisons of children from the same type of community) was present. In considering the community-context level, variance between communities still existed, but the magnitude of the heterogeneity was not significant. The final model exhibited the highest reduction of variance (PCV = 53.98%); this model included individual-, family-, and community-context levels (considering both levels of community dental service accessibility and average levels of oral health–related knowledge). In model 3, the PCV decreased to 35.61%, and only the context of individual-, family-, and community-level dental service accessibility was examined. In model 2, where no community-context cofactors were considered, and model 1, containing only individual levels, the PCVs were 20.37% and 5.52%, respectively.

**Table 4:** The multi-level analysis of children, family and community level on caries status

	Null	Model 1	Model 2	Model 3	Model 4
Caries status					
Coefficience of variation	0.1141	0.1078	0.09086	0.07347	0.05251
SE	0.03051	0.03226	0.03017	0.03231	0.02471
PCV(%)	-	5.5214724	20.368098	35.609115	53.9789658
MOR	1.3783644	1.3660354	1.3315696	1.2936929	1.24320544
ICC	0.0335184	0.0317264	0.0268748	0.0218435	0.01570975

## Discussion

To summary from the result, the caries rate of preschool children in Taiwan is still quite serious problem, and the huge difference also be found between rural and urban area. Habitual sweetened beverage, parents or caregivers who had high preventive dentistry knowledge and higher levels of education, parents or caregivers with lower levels of self-assessed oral health was a risk factor for caries status in preschool children. The community effect for oral health and prevention knowledge were also accounted for the caries status of preschool children of Taiwan.

Social, economic, and environmental factors contribute to oral health outcomes [11]. Population-based models have been applied in studies of children’s oral health, with such studies incorporating basic biological factors and other influential factors, including socioeconomic status, ethnicity, culture, stress, health behaviours, and healthcare system. Our findings indicate the key components that affect oral health in preschool children and can serve as a reference for policy makers. In Bramlett’s study, the first multilevel study of children’s oral health [12], the domain effects were significant at the child, family, and community levels; however, the relative importance of various domains was not evaluated in that study. Here, we utilised the PVC to evaluate domain effects. The higher this proportion is, the higher is the general context effect; in this study, the community effect had the greatest impact on the prevalence of caries and the frequency of regular dental visits for preschool children. The characteristics of the dental care system and the community oral health environment should be included at the community level. The effect of the community oral health environment on the oral health of preschool children seems to improve the effects of the dental care system.

Population health models are generally affected by five dimensions: genetic factors, the social environment, physical factors, health-related behaviours, and medical service [11–14]. Recent studies in public health have emphasised that the multilevel nature of determinants in population health should be considered. The multidimensional approach presented here accounts for factors not acting in isolation but rather through complex interactions [7, 15]. In a 1993 study, Reisin and Litt found that predictions could be improved by considering social and psychological factors rather than solely considering biological factors [16]. The simple, individual-based model is not adequate for examining the factors that influence health outcomes and behaviours. Multidimensional factors do not act independently; rather, they interact in complex ways to affect population health, with the effects varying by age group [12]. For preschool children, a previous study [17] provided evidence that considering social and psychological factors rather than only biological factors improved the ability to make predictions regarding the utilisation of dental services. In 2010, Bramlett applied a multilevel model to assess the factors that influence the oral health status of young children [12]. Individual-, family-, and community-level factors were considered in the model, which revealed that efforts to improve the oral health of children should be based on a multilevel model that goes beyond solely individual-level factors because comprehensive model [1, 7, 18–20]. Determinants may also change with age and developmental trajectory [20]. In a conceptual model, the direct and indirect relationships of reciprocal cause and effect can be realistically considered. The strength of such a model may be more comprehensive predictive value and superior external validity. In our study, the conceptual model was constructively applied to consider the determinants of the oral health status of preschool children and also demonstrated the greater importance of community-level factors over individual- and family-level factors, and would provide more suitable predictive results for public health research and policy making.

Socioeconomic status affects the oral-health status of children [21–23]. We used education level as a proxy for socioeconomic status. The children of two parents with a level of education above the college level had a statistically significant lower risk of caries than children with parents with levels of education below the college level. Parental education level is a crucial factor related to oral health knowledge, which influences the oral health of preschool children [7, 24]. Through proper diet habits and optimal use of preventive fluoride treatments, caries should be an entirely preventable disease [2, 25]; both these preventive behaviours must be instilled by the parents or caregivers of preschool children. We suggest that family factors have a greater impact on the risk of caries in preschool children than does the care provided in dental clinics.

Clinic-based dental intervention is unlikely to influence the behaviour of caregivers or parents [2], especially for preschool children who live in remote areas, lack dental resources, and are more likely to attend dental clinics infrequently. Community interventions can play a crucial role in the prevention of oral health problems in preschool children; by championing efforts to change family behaviour in the home, community interventions could have a greater effect on preventing caries than clinic-based interventions would [2]. Positive community-based initiatives, such as oral health promotion drives and publicising of preventive care policies, have greatly improved the oral health of children [7]. Children living in a community that values positive conceptions of oral health are more likely to have better oral health status [26, 27]. Therefore, the critical stage in the prevention of the chronic disease of dental caries is early childhood.

The consumption of sugar-sweetened beverages has been found to be associated with dental caries in preschool children. This finding is supported with growing evidence of a determinate effect of sweetened beverages on oral health [28, 29]. Restricting sugar intake should always be a critical recommendation for improving the oral health of the population [30]. The most notable predictive factor for experiencing caries in the permanent dentition is experiencing caries in the primary dentition [2]. Fluoride intervention, which includes fluoridated water, fluoridated toothpaste, fluoride supplements, and FVA, is one of the most cost-effective strategies to prevent caries. Fluoridated water and FVA are common strategies practiced in population-based preventive intervention. Pit and fissure sealants have also been demonstrated to be cost-effective [31] when applied to high-risk children [32].

The strength of this study is that a range of confounders at the individual, family, and community level was considered through appropriate multilevel analysis. Measures aiming to improve the oral health of preschool children, and in particular the prevalence of caries in these children, should target community-level factors, such as the accessibility of dental services and, especially, the improvement of oral health knowledge at the community level. Oral health education is an essential component of any oral health program. To rely solely on dentists for leading oral health education programs for children is impractical and inefficient. Training more professional oral health educators to provide additional community-based oral health promotion campaigns is critical. Such community-based programs would effectively improve the oral health knowledge and behaviours of the parents and caregivers of preschool children.

To conclude, significant variations still exist in oral health in contemporary preschool children. The most notable finding of this study is that to improve the oral health of preschool children, community-level factors should be targeted. To rely solely on dentists for leading oral health education programs for children is impractical and inefficient. Training more professional oral health educators to provide additional community-based oral health promotion campaigns is critical. We suggest training more professional oral health educators to provide more community-based oral health promotion campaigns.

### Limitations

The first limitation of this study is that our data are from a cross-sectional survey; hence, time could not be considered as a factor in our model. We suggest that longitudinal data should be collected in a future study. Potential bias also exists in this cross-sectional study because of the simultaneous collection of information concerning the outcome and independent factors. Such a study design is only able to reveal associations, not cause-and-effect relationships.

## Data Availability

The data sets used and/or analysed during the current study are available from the corresponding author upon reasonable request.
